# Characterizing exons 11 and 1 promoters of the mu opioid receptor (*Oprm*) gene in transgenic mice

**DOI:** 10.1186/1471-2199-7-41

**Published:** 2006-11-13

**Authors:** Jin Xu, Mingming Xu, Ying-Xian Pan

**Affiliations:** 1 Department of Neurology, Memorial Sloan-Kettering Cancer Center, New York, NY 10021 USA

## Abstract

**Background:**

The complexity of the mouse mu opioid receptor (*Oprm*) gene was demonstrated by the identification of multiple alternatively spliced variants and promoters. Our previous studies have identified a novel promoter, exon 11 (E11) promoter, in the mouse *Oprm *gene. The E11 promoter is located ~10 kb upstream of the exon 1 (E1) promoter. The E11 promoter controls the expression of nine splice variants in the mouse *Oprm *gene. Distinguished from the TATA-less E1 promoter, the E11 promoter resembles a typical TATA-containing eukaryote class II promoter. The aim of this study is to further characterize the E11 and E1 promoters in vivo using a transgenic mouse model.

**Results:**

We constructed a ~20 kb transgenic construct in which a 3.7 kb E11 promoter region and an 8.9 kb E1 promoter region controlled expression of tau/LacZ and tau/GFP reporters, respectively. The construct was used to establish a transgenic mouse line. The expression of the reporter mRNAs, determined by a RT-PCR approach, in the transgenic mice during embryonic development displayed a temporal pattern similar to that of the endogenous promoters. X-gal staining for tau/LacZ reporter and GFP imaging for tau/GFP reporter showed that the transgenic E11 and E1 promoters were widely expressed in various regions of the central nervous system (CNS). The distribution of tau/GFP reporter in the CNS was similar to that of MOR-1-like immunoreactivity using an exon 4-specific antibody. However, differential expression of both promoters was observed in some CNS regions such as the hippocampus and substantia nigra, suggesting that the E11 and E1 promoters were regulated differently in these regions.

**Conclusion:**

We have generated a transgenic mouse line to study the E11 and E1 promoters in vivo using tau/LacZ and tau/GFP reporters. The reasonable relevance of the transgenic model was demonstrated by the temporal and spatial expression of the transgenes as compared to those of the endogenous transcripts. We believe that these transgenic mice will provide a useful model for further characterizing the E11 and E1 promoter in vivo under different physiological and pathological circumstances such as chronic opioid treatment and chronic pain models.

## Background

Mu opioid receptors play an essential role in mediating actions of morphine and most clinical analgesic agents such as codeine, methadone and oxycodone, as well as drugs of abuse such as heroin [1,2]. Early pharmacological studies proposed several mu opioid receptor subtypes: mu_1_, mu_2 _and morphine-6β-glucuronide (M6G) [3-5]. Molecular cloning of a mu opioid receptor[6], MOR-1, has provided an invaluable tool to explore multiple mu opioid receptors at the molecular level. However, only a single copy of the mu opioid receptor (*Oprm*) gene has been identified [7-9]. Alternative pre-mRNA splicing and multiple promoters of the *Oprm *gene have been hypothesized as molecular explanations of multiple mu opioid receptors. Over the past ten years, we have extensively explored alternative splicing of the *Oprm *gene, particularly of the mouse *Oprm *gene. In addition to the rat MOR-1B and human MOR-1A reported earlier[10,11], we have identified 25 splice variants from the mouse *Oprm *gene [12-16], which are derived from various combinations of sixteen exons that span over 250 kb. We have also isolated 8 splice variants from the rat *Oprm *gene and 11 from the human *Oprm *gene [17-19]. The functional significance of these splice variants has been suggested by differences in their region-specific and cell-specific expressions, agonist-induced G protein coupling and receptor internalization[12,14,17,19-24].

The complexity of the *Oprm *gene was further demonstrated by the identification of multiple promoters. Initially, promoter activity was mapped to an approximately 1.5 kb region upstream of exon 1 (E1 promoter) in the mouse, rat and human *Oprm *genes[7-9,25]. A dual promoter model of the E1 promoter has been proposed, in which the dominant proximal promoter is approximately 500 bp apart from the distal promoter [26-28]. Within numerous putative cis-acting elements predicted from the E1 promoter region by searching transcription factor databases, several cis-acting elements such as a Sp binding sequence, a 34 bp element, a 26 bp polypyrimidine sequence, CRE, OCT1, IL-4-responsive element, NF-κappaB, SOX, and neuron-restrictive silencer element (NRSE) in the proximal or distal promoters have been identified to interact with their trans-acting partners, which positively or negatively regulate the E1 promoter activity [29-42]. For example, NRSF (neuron-restrictive silencer factor) can bind to a 21 bp NRSE element in the proximal promoter region to suppress the promoter activity [31]. Interestingly, a 10 bp Sp cis-acting element in the proximal promoter can function either as a negative element when bound to the M1 and M2 isoforms of Sp3 or as a positive element by interacting with Sp1 and Sp3[36]. The poly(C) binding proteins can interact with a 26 bp polypyrimidine sequence in the proximal promoter to enhance the transcription of MOR-1 in NMB cells[38,39]. Tumor necrosis factor can induce the mu opioid receptor gene transcription in several types of immune cells. This induction has been suggested to be mediated through induced interaction between NF-κB factor and NF-κB binding sites located in E1 promoter[41].

Recently, we have identified a new promoter (E11 promoter) in the mouse *Oprm *gene[43], which was located ~10 kb upstream of the E1 promoter. The E11 promoter controls the expression of at least nine splice variants in the mouse *Oprm *gene. A major transcription start point was mapped to a guanidine residue, 187 bp upstream from the putative translation start codon of E11[43]. A basal core region, a negative region and a positive region of the E11 promoter, were identified using sequential 5'- and 3'-deletion constructs in NIE-115 cells, a mouse neuroblastoma cell line. The basal core region contains a TATA box that can specifically bind to a TATA-binding protein (TBP) in a gel shifting assay[43]. Mutation analysis indicated that the TATA box played an essential role in the E11 promoter activity, and that a NF-1 site and a cMyc/Max site near TATA box modulated basal core promoter activity[43].

The E11 promoter differs from the E1 promoter in several aspects. First, the E11 promoter contains a TATA box that is absent in the E1 promoter. Second, the E11 promoter has one major transcription start point (tsp), while the E1 promoter contains multiple tsp. Third, although both promoters have several cis-acting elements such as CAAT box, AP-1 and NF-κB, the E1 promoter contains several GC-rich cis-acting elements like Sp1 and AP-2 that are missing in the E11 promoter. Sp1 regulates a number of TATA-less promoters. Thus, the E11 promoter appears to be a typical eukaryote class II promoter associated with RNA polymerase II, while the E1 promoter favors a "housekeeping" gene mode. Finally, the E1 promoter drives transcription of 16 variants, while the E11 promoter controls the expression of 9 other variant transcripts, three of which can translate into the same MOR-1 protein.

Most promoter studies were performed in vitro using different cell models. Although transgenic technology has been widely used to characterize opioid receptor functions in vivo, there is little information about their use in studying transcriptional regulation of the opioid receptor genes, except for the transgenic studies of the mouse kappa opioid receptor (Oprk) gene[44]. In the present studies, we establish a transgenic mouse model using a single transgenic construct that contains a 3.7 kb E11 promoter region and an 8.9 kb E1 promoter region to drive expression of two reporters, tau/LacZ and tau/GFP, respectively. This allows characterizing both E11 and E1 promoters in the same transgenic mice with X-gal staining and GFP imaging.

## Results and Discussion

### Generation of the transgenic mouse line

**Figure 1:**
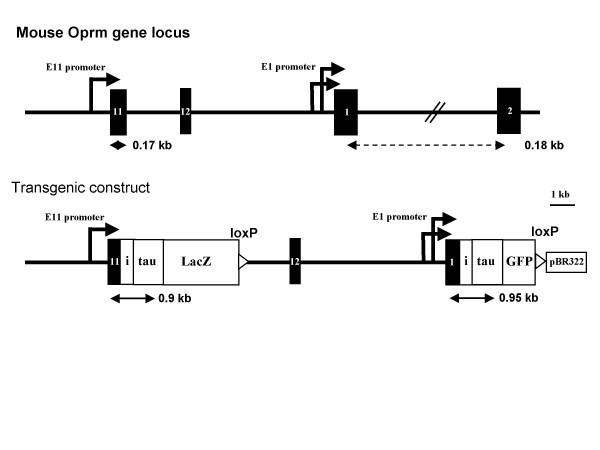
**Schematic of the transgenic construct**. Exons and introns are shown by black boxes and horizontal lines, respectively. The reporter cassettes are indicated by white boxes. The E11 and E1 promoters are shown by arrows. LoxP sites are shown by triangles. The fragments generated by the RT-PCR are indicated by double-head arrows labeled with the predicted sizes. i: IRES.

In order to study both E11 and E1 promoters in mice, we made a ~20 kb transgenic construct in a pBR322 vector (Fig. 1). The construct contained a 3.7 kb region upstream of E11 and an 8.9 kb region upstream of E1 that controlled expression of an IRES/tau/LacZ cassette and an IRES/tau/GFP cassette, respectively. Our previous studies indicated that the 3.7 kb region contained E11 promoter activity that was most evident in neuronal cells. A negative regulatory region, a basal core region containing a TATA box and a positive regulatory region were identified within a 600 bp region at the 3'-end of the 3.7 kb fragment. The 8.9 kb fragment included the dual E1 promoters that were characterized by several groups [26-28] and the regions further upstream.

The internal ribosome entry sequence (IRES) derived from the encephalomyocarditis virus can directly affect mRNA cap-independent entry of the translational apparatus in mammalian cells[45]. IRES can efficiently drive the translation of downstream tau/LacZ or tau/GFP fusion proteins in the construct. Tau is a microtubule-associated protein distributed in the axonal microtubules of neurons[46]. Recently, tau/LacZ and tau/GFP fusion proteins have been developed as a new generation of axon-targeting reporters that allow labeling subsets of neurons and their projections to study neuronal structure and function[47]. Tau/LacZ and tau/GFP reporters have been used successfully to reveal the projections of specific subsets of olfactory neurons to their axonal terminal glomeruli in knockin mouse models[48,49]. Therefore, tau/LacZ and tau/GFP cassettes were designated in the construct as reporters for E11 and E1 promoter activities, respectively. We hope that by using the axon-targeting potential of tau/LacZ and tau/GFP reporters, we are able to map E11 and E1 promoter activities not only in their expressing cell bodies but also in their projections.

**Figure 2:**
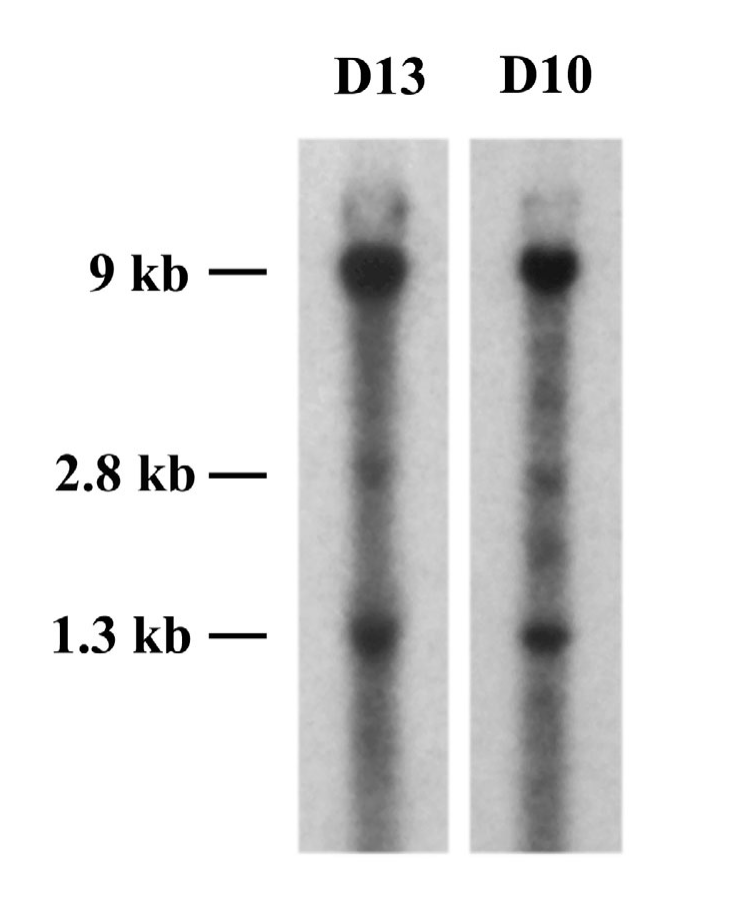
**Southern blot analysis**. Southern blot analysis was performed as described in the Methods section. Briefly, 5 μg genomic DNA from two transgenic lines, D13 and D10, was digested with BamHI, separated on 0.8% agarose gel and transferred onto a GenePlus membrane. The membrane hybridized with the GFP probe was exposed to the Kodak BioMax MS film.

Initially, six founder mice were obtained by microinjection of the construct into the pronucleus of fertilized eggs. Continuously breeding these founder mice resulted in the establishment of two transgenic lines (D13 and D10) that stably expressed the transgene. Both transgenic lines showed a similar genotyping pattern with a major ~9 kb, minor ~1.3 kb and weak ~2.8 kb BamHI digested genomic fragments in Southern blot analysis using a 0.55 kb GFP probe (Fig. 2), although the D10 line had several additional weak bands. We also compared the transgene expression in both lines by using X-gal staining and GFP imaging. The results showed that both transgenic lines displayed a similar transgene expression pattern in several brain regions (see additional file 1: Figure 1 – Comparison of tau/LacZ and tau/GFP reporter expression between two transgenic lines). We used the D13 line for the following studies. The transgenic mice were fertile and viable, with no gross morphological abnormalities.

### Expression of endogenous E11 and E1 mRNAs in wild-type C57BL/6J mice during ontogeny

**Figure 3:**
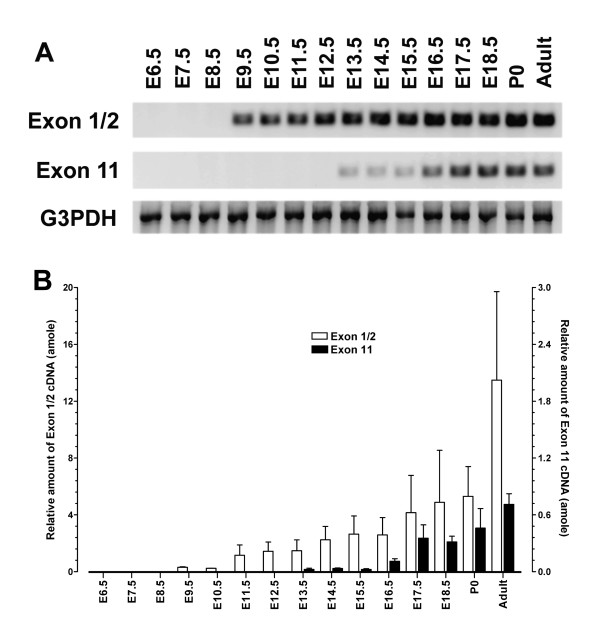
**Expression of the endogenous E11 and E1 mRNAs in wide-type C57BL/6J mice during ontogeny**. A. RT-PCR: The temporal expression of a 184 bp exons 1/2 (Exon 1/2) and a 178 bp exon 11 (Exon 11)transcripts during ontogeny of C57BL/6J mice was determined by a relative quantitative RT-PCR approach as described in the Methods section. RNA input was estimated by a parallel PCR with a pair of G3PDH primers. Agarose gel stained with ethidium bromide was photographed with FluorChem 8000 system. PCR cycles and extension time at 72°C: for Exon 1/2 primer set, 35 cycles and 20 sec; for Exon 11 primer set, 45 cycles and 20 sec; and for G3PDH primer set, 25 cycles and 2 min. B. Relative quantification: Relative band intensities from the gel were quantified with AlphaEase FC software, and converted to amole concentration based upon the linear regression equations from the linear phase of each saturation curve (see Fig. 8) using Prism 4.0. The concentration was normalized with the G3PDH concentration. Right Y axis represents the concentration of Exon 11 cDNA, and left Y axis, the concentration of Exon 1/2 cDNA. Bars represent the mean ± S.E. of the concentration from three independent experiments.

Although expression of MOR-1 or E1 promoter during ontogeny has been extensively studied using RT-PCR, in situ hybridization and in situ receptor binding[28,50-52], the expression of the E11-associated variants or E11 promoter during mouse development has not yet been reported. In order to evaluate whether the transgene in the transgenic mice has a temporal expression pattern similar to that of the endogenous E11 promoter, we first examined expression of the endogenous E11 mRNA during development. We chose C57BL/6J mice because the transgenic mice were generated by backcrossing with C57BL/6J. Since the E11 promoter controls expression of nine E11-associated variants in which E11 is the first exon[14], expression of E11 will be an assessment of the E11 promoter. We examined endogenous E11 mRNA expression using a relative quantitative RT-PCR approach with primers derived from E11. The results showed that expression of the 178 bp E11 transcript in wild-type C57BL/6J mice started at embryonic day 13.5 (E13.5) (Figs. 3A &3B). The expression levels of E11 mRNA between E13.5 and E15.5 was quite low (0.025~0.033 amole), and gradually increased to highest level in adult (0.71 amole).

We also determined expression of a 184 bp exon 1/2 (E1/2) transcript that was controlled primarily by the E1 promoter using PCRs with the same 1^st^-strand cDNAs as templates. As shown in Figs. 3A &3B, the E1/2 mRNA was detected at E9.5, which was four days earlier than the E11 mRNA (E13.5), suggesting early onset of the E1 promoter during development. The expression level of the E1/2 transcript at E9.5 and E10.5 was low (0.24~0.31 amole), slowly increased from E11.5 to P0 (1.15~5.30 amole), and reached to highest level in adult (13.48 amole). Our results are similar to those in the literature. Zhu et al. reported the detection of MOR-1 mRNA at E10.5 using an in situ hybridization approach with a [^33^P]-labeled RNA probe derived from exon 1[51], which was one day later than our result. Using a combined RT-PCR/Southern blot approach, Ko et al. detected the expression of both the exon 1/2 and the exon 1 transcripts controlled by the dual exon 1 promoters as early as E8.5[28], which was one day earlier than our result. These differences are likely the result of differences in the sensitivities of the assay methods or differences among the strains. However, all these studies observe an appearance of E1 activity at least 3 days prior to E11 activity.

The later onset of the E11 promoter activity during mouse embryonic development suggested differential regulation of the E11 and E1 promoters and raised questions regarding their functional roles in development. Further investigation and identification of the cis-acting elements and trans-acting factors involved in the E1 and E11 promoters will help illuminate the mechanisms behind such differential regulation.

The results also indicated higher levels of E1/2 mRNA than E11 mRNA during development and in the adult. For example, the E1/2 transcripts detected from E16.5 to E18.5 were 10 – 24-fold higher than the E11 transcripts (Figs. 3A &3B). In adult brain, there was an >18-fold difference between the E1/2 and E11 transcripts (Figs. 3A &3B), which is consistent with our previous observations in the adult brain tissues[14]. Designing specific primers for amplifying E1 promoter-driven transcripts is difficult since some of the E11-associated variants also contain an exon 1 region that includes part of the proximal promoter sequence. Therefore, the E1/2 primers also amplifies several E11-associated variants that contain the exons 1–2 sequence. However, since the expression level of the E11-associated transcripts was much lower than that of E1-associated transcripts, we considered the PCR products generated from E1/2 primers reasonable indications of E1 promoter activity. However, functional significance cannot be assigned only upon the overall abundance of a transcript. Regional and cell-specific expression should also be taken into account. In previous studies, we have shown regional-specific or cell-specific expression of some variants at both mRNA and protein levels[12,14,20,21]. For example, expression of MOR-1C mRNA was quite low when using the RNA from the total brain[12], but MOR-1C protein was highly expressed in certain regions such as the Lateral septum and Median eminence, when compared to the expression of MOR-1 protein[20]. It will be interesting to further explore the temporal and spatial expression patterns of these E11 mRNAs during development and in adult CNS, using an in situ hybridization approach.

### Expression of the transgene mRNAs in transgenic mice during ontogeny

**Figure 4:**
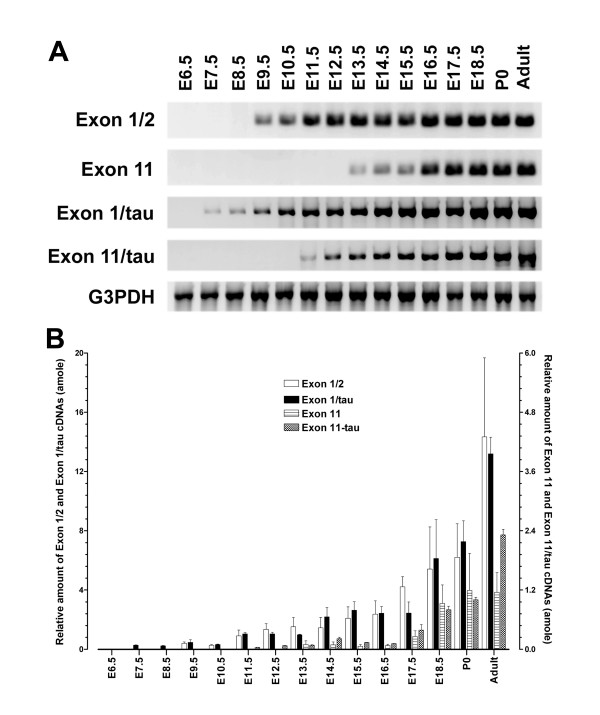
**Expression of the endogenous E11 and E1 and thetransgene mRNAs in the transgenic mice during ontogeny**. A. RT-PCR:The temporal expression of a 184 bp exons 1/2 (Exon 1/2) and a 178 bp exon 11 (Exon 11) transcripts during ontogeny of transgenic mice was determined by the same relative quantitative RT-PCR approach used in C57BL/6J mice. A 0.95 kb Exon 1/tau transcript and a 0.9 kb Exon 11/tau transcript were determined by a similar RT-PCR approach using the same first-strand cDNAs as templates, as described in the Methods section. RNA input was estimated by a parallel PCR with a pair of G3PDH primers. Agarose gel stained with ethidium bromide was photographed with FluorChem 8000 system. PCR cycles and extension time at 72°C: for Exon 1/2 primer set, 35 cycles and 20 sec; for Exon 11 primer set, 45 cycles and 20 sec; for Exon 1/tau primer set, 35 cycles and 2 min; for Exon 11/tau primer set, 45 cycles and 2 min, and for G3PDH primer set, 25 cycles and 2 min. B. Relative quantification: Relative band intensities from ethidium bromide-stained gel were quantified with AlphaEase FC software, and converted to amole concentration based upon the linear regression equations from the linear phase of each saturation curve (see Fig. 8) using Prism 4.0. The concentration was normalized with the G3PDH concentration. Right Y axis represents the concentration of Exon 11 and Exon11/tau cDNAs, and left Y axis, the concentration of Exon 1/2 and Exon 1/tau cDNAs. Bars represent the mean ± S.E. of the concentration from three independent experiments.

In order to compare directly the endogenous E11 and E1 promoters with the transgenes, we examined the expression of endogenous E11 and E1/2 mRNA during development in the transgenic line using the same RT-PCR approach. Since the 3'-end of exon 11 coding sequence was not included in the transgenic construct, the E11 primer used for the endogenous gene would not amplify the transgene. As shown in Figs. 4A &4B, we observed the same expression profiles of E11 and E1/2 mRNA in the transgenic mice as seen in the wild-type C57BL/6J mice. The appearance of E11 and E1/2 mRNA was at E13.5 and E9.5, respectively. The expression level of E11 transcript between E13.5 and E15.5 was also low (0.06~0.10 amole), and gradually increased to the highest level in adult brain (1.15 amole).

We then examined temporal expression of the transgenes using the same RNAs in the RT-PCR with primers specifically designed for the transgenes. The E11/LacZ mRNA, which is controlled by the 3.7 kb E11 promoter in the transgenic construct, was detected as early as E11.5, which was two days earlier than the onset of endogenous E11 mRNA (Fig. 4). The expression level of the E11/tau transcript at E11.5 was quite low (0.04 amole), and slowly increased to higher levels in adult brains (2.31 amole). The E1/tau mRNA, which was transcribed through the 8.9 kb E1 promoter in the transgenic construct, was detected at E7.5, which was also two days earlier than the endogenous E1/2 mRNA (E9.5). The expression level of E1/tau transcript between E11.5 and E13.5 was low (0.98~1.36 amole), and gradually increased to higher levels in adult brain (13.19 amole). The expression level of both E11/tau and E1/tau transcripts were comparable to those of the endogenous transcripts (Fig. 4B). These results confirm the same relative temporal appearance of the E11 and E1 promoters in the transgenic construct, with the E1/tau mRNA appearing 4 days prior to the E11, similar to that of the endogenous promoters. The earlier appearance of the transgene mRNA may be due to a variety of factors. Distal control elements may be lacking in the transgenic construct, or adjacent elements of the integration site may be influencing expression. Nevertheless, the ability of the transgene to maintain the four day difference in expression between the two promoters implies that the transgenic mice provide a relevant model to study the E11 and E1 promoters in vivo.

### Distribution of tau/LacZ and tau/GFP reporters in the transgenic mouse central nervous system

We next examined brain distributions in the transgenic mice of the two reporter proteins, tau/LacZ and tau/GFP, which were controlled by the transgenic E11 and E1 promoters, respectively, using X-gal staining and GFP imaging. X-gal staining for tau/LacZ reporter activity, which is under the control of the E11 promoter, was examined in brain sections after perfusion, whereas GFP imaging for the tau/GFP reporter used fresh-frozen sections. Sections with similar regions were selected for comparison.

**Figure 5:**
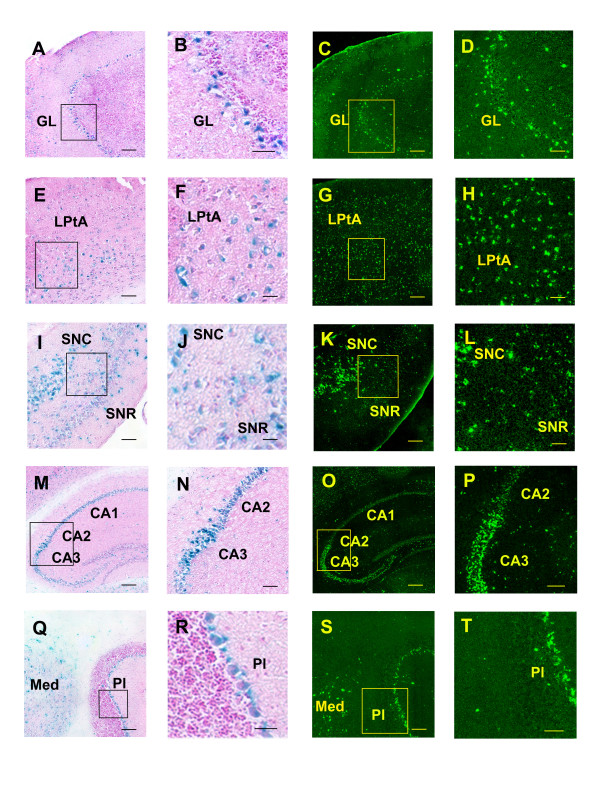
**Distribution of the tau/LacZ and tau/GFP fusion proteins in the transgenic mouse brain**. The left two columns shows X-gal staining and the right two columns, GFP imaging. The second column B, F, J, N and R is the higher power of boxes indicated in the first column A, E, I, M and Q, respectively. The last column D, H, L, P and T is the higher power of boxes shown in the third column C, G, K, O and S, respectively. GL, glomerular layer of the olfactory bulb; LPtA, lateral parietal association cortex; SNC, substantia nigra, compact; SNR, substantia nigra, reticular; CA1, field CA1 of hippocampus; CA2, field CA2 of hippocampus; CA3, field CA3 of hippocampus; Med, medial cerebellar nucleus; Pl, purkinje cell layer. Scale bar = 250 μm (A, C, E, G, I, K, M, O, Q and S) or 50 μm (B, D, F, H, J, L, N, P, R and T).

**Figure 6:**
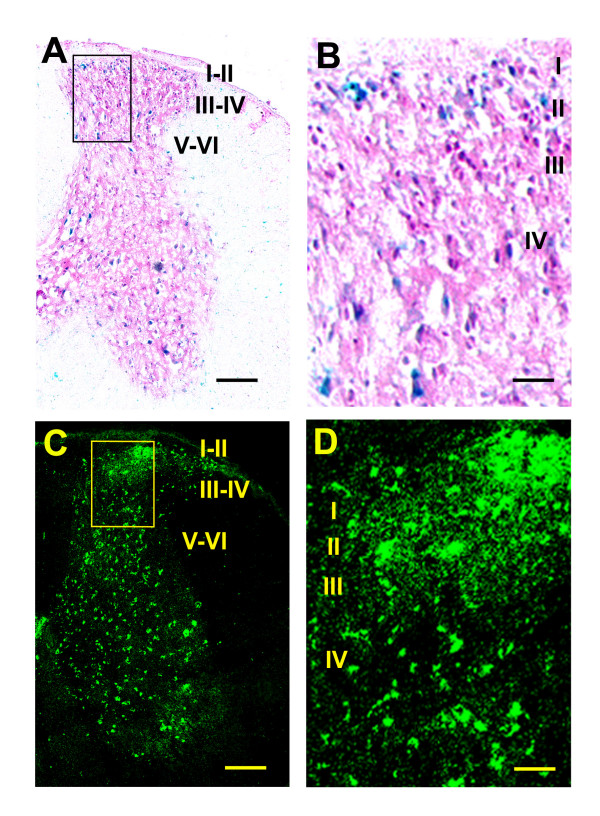
**Distribution of the tau/LacZ and tau/GFP fusion proteins in the transgenic mouse spinal cord**. A & B are X-gal staining, and C & D, GFP imaging of lumbar sections. B and D are the higher power of boxes shown in A and C, respectively. I – VI, laminae layers. Scale bar = 250 μm (A and C) or 50 μm (B and D).

**Figure 7:**
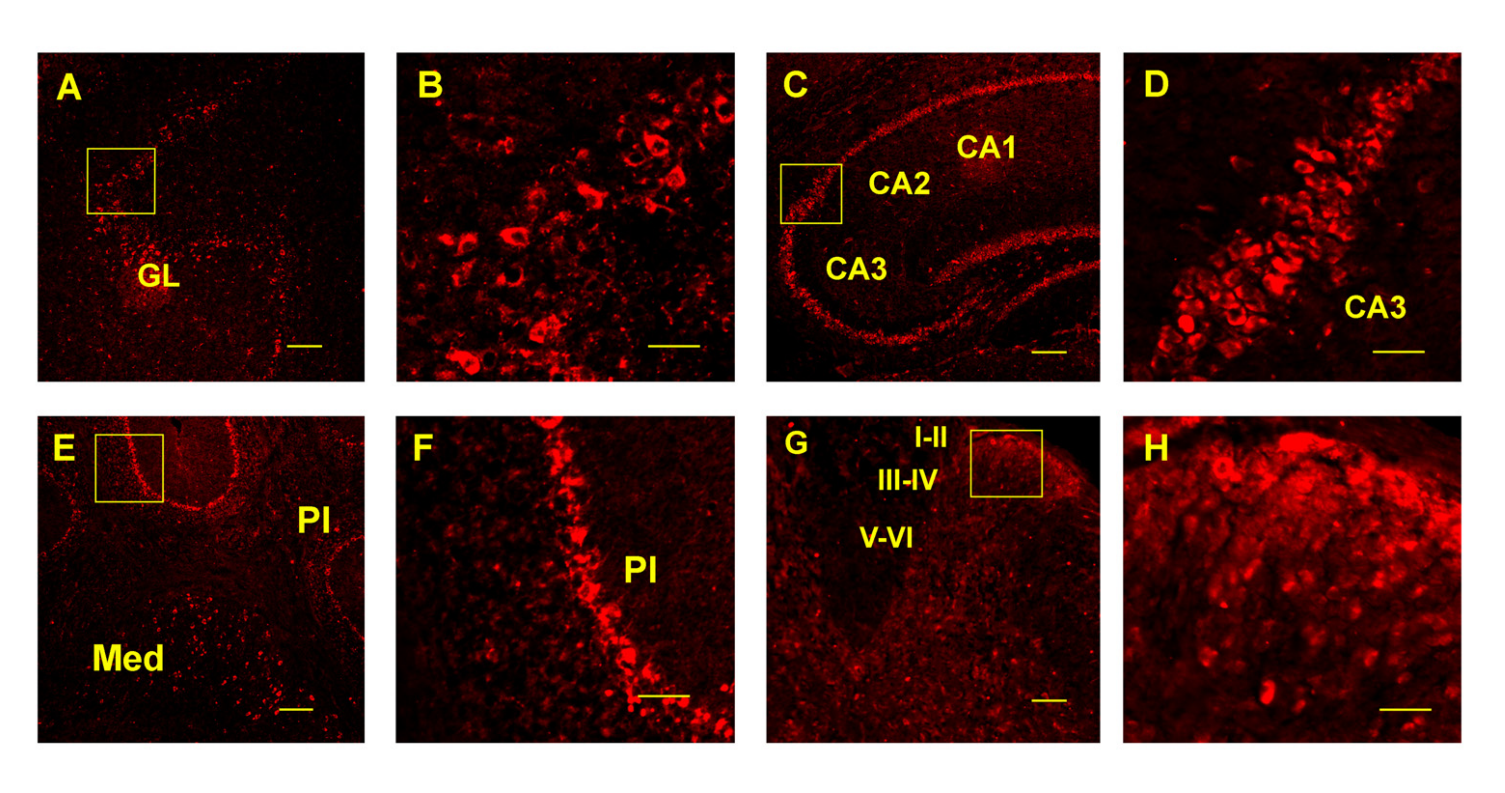
**Distribution of E4-LI in the transgenic mouse CNS**. Brain and spinal cord sections were labeled with the exon 4-specific antibody, as described in the Methods section. B, D, F and H are the higher power of boxes shown in A, C, E and G, respectively. GL, glomerular layer of the olfactory bulb; CA1, field CA1 of hippocampus; CA2, field CA2 of hippocampus; CA3, field CA3 of hippocampus; Med, medial cerebellar nucleus; Pl, purkinje cell layer. I-VI, laminae layers of lumber section. Scale bar = 250 μm (A, C, E, and G) or 50 μm (B, D, F and H).

We observed that both reporter activities were widely distributed in a number of brain regions, such as the piriform cortex, olfactory bulb, striatum, hippocampal formation, thalamus, substantia nigra, hypothalamus, brainstem, purkinje cells and spinal cord (Figs. 5 &6 and Table 1). The distribution pattern of the tau/GFP reporter was generally comparable to that obtained from in situ hybridization studies using probes derived from MOR-1 (exons 1–4) sequences [53-55] and to immunohistochemical studies using polyclonal antibodies generated against the last 12 or 15 amino acids of C-terminus of MOR-1 in rat [20,56,57]. However, these published data were mainly derived from rat brain and spinal cord sections. To directly compare tau/GFP reporter with MOR-1-LI, we examined MOR-1-LI in brain and spinal cord of the transgenic and wide-type C57BL/6J mice with a polyclonal antibody against the last 15 amino acids of MOR-1. Since the 12 of 15 amino acids are derived from exon 4, we also refer this antibody immunoreactivity to exon 4-like immunoreactivity (E4-LI). We observed similar E4-LI distributions in both the transgenic and wide-type C57BL/6J mice (data not shown), suggesting that the transgene had little effect on E4-LI distribution. As shown in Fig. 7 and Table 1, E4-LI in transgenic mice also was similar to studies in the rat [20,56,57]. For example, the hippocampal formation and the superficial laminae of the spinal cord were intensely labeled in both mice and rat.

**Table 1:** Distribution of E11 and E1 promoter activities in the transgenic CNS

**Structure**	**tau/LacZ (E11 promoter)**	**E11-LI**	**tau/GFP (E1 promoter)**	**E4-LI**
Cerebral cortex				
Piriform cortex	+++	-	+++	++
Lateral parietal association cortex	+	-	++	+
Primary motor cortex	+	-	+	+
Olfactory system				
Olfactory bulb	++	-	+	+
Olfactory tubercule	+	++	+/-	+
Striatum	+	+	+	++
Nucleus accumbens	+	+	+	++
Globus pallidus	+	+	+	+
Ammon's horn				
CA1	++	-	+/-	+
CA2	++	-	+/-	+
CA3	+++	-	+++	++
Pyramidal cell layer	+	-	+	+
Polymorph layer dentate gyrus	++	-	+	+
Subthalamic nucleus	+/-	-	+++	++
Zona incerta	+	-	+	-
Thalamus				
Laterodorsal thalamic nucleus	++	-	+/-	+
Lateral posterio thalamic nucleus	++	-	+/-	+
Ventral posteromedial thalamic nucleus	+	-	++	+/-
Ventral posterolateral thalamic nucleus	+	-	++	+/-
Hypothalamus	+	-	+	+
Substantia nigra, reticular	++	++	+/-	+/-
Substantia nigra, compact	+++	-	++	+
Locus coeruleus	+	-	+	++
Cerebellum				
Purkinje cell	+++	-	+++	+++
Medial cerebellar nucleus	++	-	++	++
Brainstem	++	-	++	++
Spinal cord				
Laminae I-II	+	-	+++	+++
Laminae III-IV	+	-	++	+
Laminae V-VI	+	-	++	+
Laminae VII-VIII	+	-	++	+
Laminae IX	+	-	++	+

E11 promoter activity indicated by X-gal staining of tau/LacZ fusion protein, E1 promoter activity indicated by GFP imaging of tau/GFP fusion protein and exon 4-like immunoreactivity (E4-LI) shown by immunostaining with an E4 antibody in the selected areas of the transgenic mice are presented. The intensity of X-gal staining, green fluorescent protein signal and Alex568 fluorescence of MOR-1-LI was visually estimated as high (+++), moderate (++), low (+), very low (+/-), and negative (-). Exon 11-like immunoreactivity (E11-LI) is from the literature[58].

The distribution of tau/GFP reporter mimicked that of E4-LI in transgenic mice (Figs. 5, 6, 7 and Table 1), including the olfactory bulb, piriform cortex, thalamus, cerebellum and spinal cord. Since E4-LI represented the MOR-1 expression mainly controlled by the E1 promoter, these results suggested that the tau/GFP reporter reasonably resembled the endogenous E1 promoter expression. However, there were some subtle differences. For example, tau/GFP reporter was primarily observed in the CA3 region of the hippocampus, while the E4-LI was distributed throughout the CA1, CA2 and CA3 regions. In the spinal cord, both tau/GFP reporter and E4-LI were heavily detected in the superficial laminae, but the tau/GFP reporter seemed to label more large neurons in the deep laminae. Different targets designated by these two methods may explain these disparities. The tau/GFP reporter was driven by the transgenic E1 promoter, but E4-LI only detected variants expressing the exon 4 sequences. There are 14 additional variants under the control of the E1 promoter that do not contain exon 4. Therefore, the labeling in the deep laminae of the spinal cord by the tau/GFP reporter may represent expression of those E1 promoter-driven variants other than MOR-1 itself. On the other hand, there are three E11 promoter-driven transcripts (mMOR-1 H, mMOR-1I and mMOR-1J) that contain exon 4 and that can generate the same identical protein as MOR-1. Thus, more labeling by the exon 4 antibody and less labeling by tau/GFP reporter in the CA1 and CA2 regions of the hippocampus may indicate that E4-LI seen in the CA1 and CA2 regions reveal transcripts driven by the E11 promoter. This possibility is supported by the expression of the tau/LacZ reporter driven by the transgenic E11 promoter in the CA1 and CA2 regions (Fig. 5M–N). However, this possibility remains speculative.

We also observed the expression of tau/LacZ reporter in various CNS regions (Figs 5 &6 and Table 1). Previously, we examined the distribution of exon 11-like immunoreactivity (E11-LI) in the mouse CNS with a polyclonal antibody generated against a 22-residue peptide from exon 11 [58]. E11-LI was observed primarily in the olfactory tubercle, striatum, globus pallidus and substantia nigra. Tau/LacZ reporter was also expressed in these regions. However, the E11-LI had a very limited distribution in the CNS in contrast to the wide distribution of tau/LacZ reporter (Table 1). There are several potential explanations. First, the tau/LacZ reporter measures the activity of the transgenic E11 promoter, and resembles the expression of all E11-associated variants. Since several E11-associated transcripts (MOR-1 H, MOR-1I and MOR-1J) can produce the same MOR-1 protein lacking the exon 11 epitope, the exon 11 antibody was incapable of detecting these proteins. Therefore, the tau/LacZ reporter reflects a broader range of variants than those identified with only the exon 11 antibody. Secondly, mismatches between the distributions of mRNA and protein within the brain are not uncommon. Mismatches have been observed for mu opioid receptors when comparing *in situ *hybridization, immunoreactivity and receptor binding between receptor mRNA and protein expression [59,60]. We also observed some mismatches between the mRNA and protein distribution of the E11-associated variants[14,58]. One possible explanation is that receptor proteins may be synthesized in cell bodies from one region and then transported to their terminals located in the other region, whereas mRNA is primarily localized in the cell body. The exon 11 antibody only detected the exon 11-associated proteins. If the exon 11 expressing proteins are transported away from the cell body, the antibody may only label the projection areas and not the cell bodies themselves. Thirdly, these differences may be dependent upon different assay sensitivities. For example, X-gal staining might be more sensitive than the exon 11 immunohistochemistry. Although tau/LacZ reporter was anticipated to resemble E11 promoter activity, the complete distribution patterns of the endogenous E11 promoter activity at both mRNA and protein levels have not been determined. However, a tau/LacZ reporter in our exon11 knockin/knockout mice, in which the exon 11 coding and adjacent intron regions were replaced by an IRES/tau/LacZ/neo cassette through homologous recombination, displayed a distribution pattern very similar to that of the transgenic mice (Xu, Rossi, Matulonis, Pasternak and Pan, unpublished data), further supporting the relevance of the current model.

Although both tau/LacZ and tau/GFP reporters were widely distributed in various CNS regions such as the piriform cortex, olfactory bulb, striatum, hippocampal formation, thalamus, substantia nigra, hypothalamus, brainstem, purkinje cells and spinal cord, the distribution patterns of the two reporters differed in certain brain regions. For example, in the lateral parietal association cortex, tau/GFP reporter was expressed over multiple layers, while tau/LacZ reporter was distributed in the middle layers only (Fig. 5E–H). In the hippocampal formation, tau/LacZ activity was observed in the pyramidal cell layer throughout the CA1, CA2 and CA3 regions, but tau/GFP activity was limited to the CA3 region (Fig. 5M–P). In the pars reticulate of the substantia nigra, the tau/LacZ reporter appeared to be expressed in more neurons than did the tau/GFP reporter (Fig. 5I–L). These differential distributions provided in vivo evidence that the E11 and E1 promoters were differentially regulated in these CNS regions.

Interestingly, both tau/LacZ and tau/GFP expression were quite prominent in Purkinje cells and in the medial cerebellar nucleus, suggesting potential roles in motor actions (Fig. 5Q–T). Although a number of studies using in situ hybridization and immunohistochemistry failed to detect MOR-1 expression in the cerebellum [20,56,57], others observed MOR-1 expression at both the mRNA and protein levels [12,13,55,61-63]. For example, Mrkusich et al. demonstrated abundant expression of both MOR-1 mRNA by fluorescent in situ hybridization and MOR-1 protein by immunostaining with an exon 4-specific antibody in the rat cerebellum, particularly within Purkinje cells and the deep cerebellar nuclei[61]. Using an in situ hybridization approach with a ^35^S-labeled 777 bp MOR-1 probe, Kaufman et al. showed moderate labeling in the medial and interposed deep nuclei in the mouse cerebellum[55]. MOR-1C, MOR-1F and MOR-1 mRNAs were also detected in the mouse cerebellum by a RT-PCR approach [12,13]. Moreover, mMOR-1B4-like immunoreactivity was densely distributed in Purkinje cells and the deep cerebellar nuclei[62]. The expression of tau/LacZ and tau/GFP reporters in Purkinje cells and medial cerebellar nucleus of our transgenic mice supported these positive findings.

In the spinal cord, both reporters were mostly distributed in the lumbar and sacral segments as compared with the cervical and thoracic segments. In the lumber segments, the expression of tau/GFP reporter appeared higher than that of tau/LacZ reporter throughout all laminae (Fig. 6). Tau/GFP reporter was intensively expressed in the superficial laminae, and also scattered in a number of motor neurons in the deeper laminae (Fig. 6C–D). Tau/LacZ reporter was also mainly distributed in laminae I-II, but had less intense labeling than tau/GFP reporter. Tau/LacZ was also observed in the larger motor neurons in the deeper laminae (Fig. 6A–B).

## Conclusion

We have established a transgenic mouse line using a ~20 kb transgenic construct in which the 3.7 kb E11 promoter region and the 8.9 kb E1 promoter region control expression of the tau/LacZ and tau/GFP reporters, respectively. This transgenic model permits studying the E11 and E1 promoters of the mouse *Oprm *gene in vivo. Temporal and spatial expressions of these reporters were similar to that of the endogenous promoters, suggesting the reasonable relevance of this transgenic model. Although the E11 transcript was expressed at lower levels, as determined by RT-PCR, differential expression of E11 and E1 promoters during embryonic development and in various CNS regions provides another mechanism for transcriptional regulation of the *Oprm *gene. We anticipate that these transgenic mice will also offer a useful in vivo model to study the E11 and E1 promoters under different physiological and pathological conditions such as chronic opioid treatment and chronic pain models.

## Methods

### Transgenic construct

A 1.1 kb tau fragment was amplified by PCR with a sense primer containing a NcoI site (5'-GAA CCA CCA TGG CTG AGC CCC GCC AGG AGT TCG ACG-3') and an antisense primer containing a BamHI site (5'-GAT GGG ATC CCC GGA CAC GAT CTC CGC CCC GTG GTC GGT CTT GG-3') using the first-cDNA synthesized from Bovine brain mRNA (ClonTech) as template. A 3.1 kb LacZ fragment was generated by PCR with a sense primer containing a BamHI site (5'-CGG GGA TCC CGT CGT TTT ACA ACG TCG TG-3') and an antisense primer containing a loxP sequence with a FseI and a AflII site (5'-GAT TGC CTT AAG GGC CGG CCA TAA CTT CGT ATA GCA TAC ATT ATA CGA AGT TAT CCC CCC TGC CCG GTT ATT ATT ATT TTT GAC ACC-3') using pMC1871 vector (Pharmacia) as template. A 0.7 kb GFP fragment was produced by PCR with a sense primer containing a BamHI site (5'-GAC GGG GAT CCC GTG AGC AAG GGC GAG GAG CTG TTC-3') and an antisense primer containing a loxP sequence with a PacI and a AflII site (5'-GAT TGC CTT AAG TTA ATT AAG ATA ACT TCG TAT AGCATA CAT TAT ACG AAG TTA TAG AGT CGC GGC CGC TTT ACT TGT AC-3') using pEGFP-1 vector (ClonTech) as template. In order to construct IRES/tau/LacZ and IRES/tau/GFP cassettes, the 0.6 kb EcoRI/NcoI IRES fragment cut from a IRES/tau/LacZ/LTNL cassette, a kind gift from Dr. Peter Mombaerts, the Rockefeller University, who has productively used it to study the olfactory system[48], the 1.1 kb NcoI/BamHI tau fragment and the 3.1 kb BamHI/AflII LacZ or the 0.7 kb BamHI/AflII GFP fragment were sequentially subcloned into the EcoRI/AflII sites pcDNA3.1 vector containing an AscI site in the polylinker, to construct IRES/tau/LacZ-pcDNA3 and IRES/tau/GFP-pcDNA3 vectors, respectively, in which the tau was in frame with LacZ or GFP to make tau/LacZ or tau/GFP fusion protein. Then a 3.7 kb XbaI/XhoI fragment containing the exon 11 and its upstream promoter region amplified by PCR with a sense primer containing a XbaI site (5'-GAC TCT AGA GCA TTG TGG TAT GCC ATT ACT ATC CAT TTA C-3') and an antisense primer containing a XhoI site (5'-GAC CTC GAG GAA AGC TTC CAT CAT CGG CCC AGA TCC-3') was subcloned into the XbaI/XhoI sites of IRES/tau/LacZ-pcDNA3 vector to construct E11P/IRES/tau/LacZ-pcDNA3 vector. For subsequent cloning, the IRES/tau/GFP cassette digested with NheI/AflII from IRES/tau/GFP-pcDNA3 was subcloned into NheI/AflII sites of pBR322 vector containing an unique polyliner (PacI-AscI-FseI-NheI-AflII) to construct IRES/tau/GFP-pBR322 vector. A 8.9 kb FseI/NheI genomic fragment containing exons 12 and 1 and exon 1 promoter region generated by PCR from a mouse genomic BAC clone[14] with a sense primer including a FseI site (5'-GAC CAC TTA GGC CGG CCA AAA GCT CAG ACA GAG AGA TGG AAA TCA AGA GGG GAA GAG-3') and an antisense primer including a NheI site (5'-GAC ACT GCT AGC TGC TGT CCA TGG TTC TGA ATG CTT GCT GCG GAC TCG GTA GGC-3') was then subcloned into the FseI/NheI sites of IRES/tau/GFP-pBR322 vector to construct E1P/IRES/tau/GFP-pBR322 vector. Finally, the E11P/IRESP/tau/LacZ cassette digested with AscI/FseI from the E11P/IRES/tau/LacZ-pcDNA3 vector was subcloned into the AscI/FseI sites of E1P/IRES/tau/GFP-pBR322 vector to make the final construct with a ~20 kb insert in pBR322 vector (Fig. 1). Two loxP sites were included in the construct, one located at downstream of the tau/LacZ cassette and the other at downstream of the tau/GFP cassette, which allows Cre-mediated recombination to remove E1 promoter and tau/GFP cassette. All PCR products and cloning joints were confirmed by DNA sequencing with appropriated primers.

### Generation of transgenic mice

DNA microinjection and embryonic manipulations were performed in Transgenic Core Facility of MSKCC using standard procedures[64]. Briefly, the linearilized vector with AscI was injected into pronuclei in one-cell eggs that were isolated from superovulated C57BL/6J females mated with CBA/Ca males (B6CBA/Ca F1). About 20–30 injected ova were transplanted into the oviduct of a pseudopregnant B6CBA/Ca F1 recipient that carries the eggs to term. Genotyping was performed by Southern blot analysis of genomic DNA isolated from offspring tails using a 0.55 kb GFP fragment as probe. The integrity of the transgene in the transgenic lines was confirmed by PCRs with appropriate primers. The transgenic lines with stable transgene transmission were established by continuously backcrossing the founder with C57BL/6J mice.

### Southern blot analysis

Genomic DNA was isolated from mouse tails using a DNeasy kit (Qiagen) by following the manufacture protocol. 5 μg genomic DNA was digested with BamHI, separated on 0.8% agarose gel, and transferred onto GenPlus membrane (NEB). The membrane was hybridized with a ^32^P-labeled 0.55 kb GFP probe generated by PCR with appropriate primers. After washing, the membrane was exposed to Kodak BioMax MS film.

### Tissue preparation and RNA isolation

All animal studies were conducted in accordance with the Guide for the care and use of laboratory animals and the Animal Welfare Act, and reviewed and approved by the IACUC. Mouse embryos were collected at various developmental stages between days 6.5 and 18.5 (E6.5–E18.5) from wild-type C57BL/6J and transgenic females based upon the standard procedures as described[64]. Morning on the day of detecting a vaginal plug was designated as day 0.5. The day of birth was designated as P0. All embryos were dissected free of maternal tissue. 20 – 40 embryos from E6.5 to E9.5, 10 – 15 embryos from E10.5 to E12.5, 5 – 8 embryos from E13.5 to E15.5, and 2 – 3 embryos from E16.5 to P0 were collected for RNA extraction. The embryos from E6.5 to E15.5 were pooled, while individual embryos from the rest stages were used. Whole embryo tissue from E6.5 to E10.5 and brain from E11.5 – E18.5 embryos, P0 and adult mice were used for extracting total RNA using the guanidinum thiocyanate phenol-chloroform extraction method[65].

### Reverse-transcription-polymerase chain reaction (RT-PCR)

**Figure 8:**
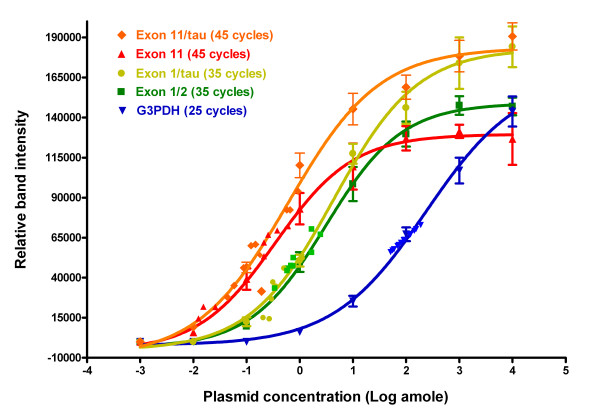
**PCR saturation studies**. Saturation curve for each set of primers with different PCR cycles was established as described in the Methods section. In brief, PCRs with the indicated primer sets and cycle numbers were performed by using templates from a series of dilution of the known concentration of plasmids containing the target sequences. The plasmid concentration was ranged from 0.001, 0.01, 0.1, 1, 10, 100, 1000 and 10,000 amole. Relative band intensities from the ethidium bromide-stained gel were quantified with AlphaEase FC software. The linear phase of amplification for Exon 11 and Exon 11/tau targets (0.01 – 10 amole), Exon 1 and Exon 1/tau targets (0.1 – 100 amole), and G3PDH target (1~10,000 amole) was used to establish the linear regression equation using Prism 4.0. The sample concentration was calculated from the linear regression equation. The curves represented from three to six independent experiments. Each individual spot represents a single sample from the indicated primer set. Only part of the samples is displayed.

6 μg of total RNA was treated with DNAse I using the Turbo DNAse-free reagents (Ambion) to remove potentially contaminated genomic DNA, and reverse-transcribed with random hexamers and Superscript II reverse transcriptase (Invitrogen) as previously described[12,14]. The RT product was treated with RNase H to remove RNA complementary to the first-strand cDNA. The first-strand cDNA synthesized from 0.3 μg RNA was then used as a template in PCRs to amplify a 178 bp endogenous E11 fragment with a sense primer (5'-GTC CTT GAG AAT GGA GAG GAT CAG CAA AGC-3') and an antisense primer (5'-CTG AGG TAA CTC TTC CCC TCT TGA TTT C-3'), a 184 bp endogenous exon 1/2 fragment with a sense primer (5'-GCC CTC TAT TCT ATC GTG TGT GTA GTG G-3') and an antisense primer (5'-CCA CGT TCC CAT CAG GTA GTT AAC AC-3'), a 0.9 kb E11/tau fragment with a sense primer from E11 (5'-GAT CTG GGC CGA TGA GGA AGC TTT CTC-3') and an antisense primer from tau (5'-GTC GGA GTG CTC TTA GCA TCA GAG GTT TCA G-3') and a 0.95 kb E1/au fragment with a sense primer from E1 (5'-GCT TGT CCT TGT AAG AAA CTG ACG GAG CCT AG-3) and antisense primer from tau (5'-GTC GGA GTG CTC TTA GCA TCA GAG GTT TCA G-3'). RNA loading was estimated by a parallel PCR with a pair of glyceraldehyde-3-phosphate dehydrogenase (G3PDH) primers (ClonTech). A negative control template obtained from a RT reaction without adding Superscript II was also used in the PCRs with the same pairs of primers. PCR reactions were carried out using Platinum Taq DNA polymerase (Invitrogen) for 25 – 45 cycles after 2 min at 94°C, each cycle consisting of a 20 sec denaturing step at 94°C, a 20 sec annealing step at 65°C and a 20 – 120 sec extension at 72°C. The PCR products were analyzed in the same 2% agarose gel, stained with ethidium bromide and photographed with FluorChem 8000 Image system (Alpha Innotech). The relative band intensities from the gel were quantified with AlphaEase FC software. The amplified fragments were confirmed by sequencing. In order to quantify the amount of transcripts from different target with a wide range of abundance, various PCR cycle numbers and extension times were adjusted to amplify cDNA at linear phase. For lower abundant E11 and E11-tau transcripts, we used 45 cycles/20 sec extension and 45 cycle/120 sec extension, respectively. For medium abundant E1/2 and E1-tau transcripts, we amplified for 35 cycles/20 sec and 35 cycles/120 sec, respectively. For higher abundant G3PDH transcript, we only used 25 cycles/120 sec extension. We also obtained the same results from PCRs with amplification for 50 cycles except that the products seemed saturated (data not shown). To quantify the transcripts more accurately, we first established the saturation curve with the known concentration of plasmid containing the target sequences with each pair of primers. The plasmid concentration was ranged from 0.001, 0.01, 0.1, 1, 10, 100, 1000 and 10,000 atto mole (amole). As shown in Fig. 8, the linear phase of amplification for E11 and E11/tau sequences, E1 and E1/tau sequence, and G3PDH sequence was 0.01~10 amole, 0.1~100 amole, and 1~10,000 amole, respectively. The PCR products at higher template concentrations under certain PCR cycle conditions tended to be saturated. The linear regression equation for each curve was calculated from the linear phase of amplification using Prism 4.0. The sample concentration was calculated based upon the linear regression equation, and normalized with the G3PDH concentration. As expected, most amplification for various samples and targets were maintained in the linear phase of amplification (Fig. 8).

### X-gal staining and GFP imaging

Mice were anesthetized with Ketamine-xylazine and transcardially perfused with 4% paraformaldehyde in PBS. Brain and spinal cord tissues were dissected, post-fixed with 4% paraformaldehyde in PBS for 2 hrs, incubated with 25% sucrose in PBS at 4°C until tissue sank to bottom, and frozen in Tissue-Tek OCT (Miles, Elkhart, IN). 10 micrometer frozen sections were cut on a Leica Cryomicrotome and used for X-gal staining as follows. The sections were washed with PBS and incubated with X-gal staining solution containing 2 mM MgCl_2_, 10 mM potassium-ferricyanide, 10 mM potassium-ferrocyanide and 1 mg/ml X-gal in PBS for 18 hrs at 37°C. After washing with PBS, the sections were counterstained with Nuclear Fast Red (Vector Lab), dehydrated and mounted with Permount (Fisher). 10 micrometer frozen sections cut from fresh frozen tissues were directly used for GFP imaging. All sections were examined and photographed with a Zeiss Axioplan 2 Imaging microscopes with ApoTome™ or a Zeiss Axiovert 200 M with a MetaMorph Imaging System.

### Immunohistochemistry

10 micrometer frozen sections from the perfused tissues prepared from the same procedure for X-gal staining were used for immunostaining. After blocking in a blocking solution containing 5% normal goat serum (NGS) in PBS for 1 hr at room temperature, the sections were incubated with a rabbit polyclonal antibody generated against the last 15 amino acids of MOR-1 (Neuromics, Minneapolis, MN) in PBS containing 3% NGS (1:50 dilution) overnight at 4°C, washed three times with PBS, blocked in the above blocking solution for 30 min at room temperature, and then incubated with an Alexa Fluor 568 conjugated goat anti-rabbit IgG (Molecular Probe) (1:500 dilution). After washing three times with PBS, the sections were cover-slipped with Vectashield mounting medium (Vector Laboratories) and examined and photographed with a Zeiss Axioplan 2 Imaging microscopes with ApoTome™.

## Authors' contributions

JX and MMX participated in construction of the transgenic construct, generation of transgenic mice, and analyses of mRNA and reporter expression. YXP designed, coordinated and drafted the manuscript. YXP also performed imaging analysis. All authors approved the manuscript.

## Supplementary Material

Additional file 1 Figure 1 – **Comparison of tau/LacZ and tau/GFP reporter expression between two transgenic lines**. The left two columns shows X-gal staining and the right two columns, GFP imaging. A, C, E, G, I and K are obtained from D13 line and are the same images shown in Fig. 5. A, C, M, O, Q and S, respectively. B, D, F, H, J and L are derived from D10 line. GL, glomerular layer of the olfactory bulb; CA1, field CA1 of hippocampus; CA2, field CA2 of hippocampus; CA3, field CA3 of hippocampus; Med, medial cerebellar nucleus; Pl, purkinje cell layer. Scale bar = 250 μm (A, C, E, G, I and K) or 50 μm (B, D, F, H, J and L). Click here for file
