# Supplementary figures and images for: Characterizing exons 11 and 1 promoters of the mu opioid receptor (Oprm) gene in transgenic mice
Source: BMC Mol Biol. 2006 Nov 13;7:41. doi: 10.1186/1471-2199-7-41 (PMC1657025; doi:10.1186/1471-2199-7-41)

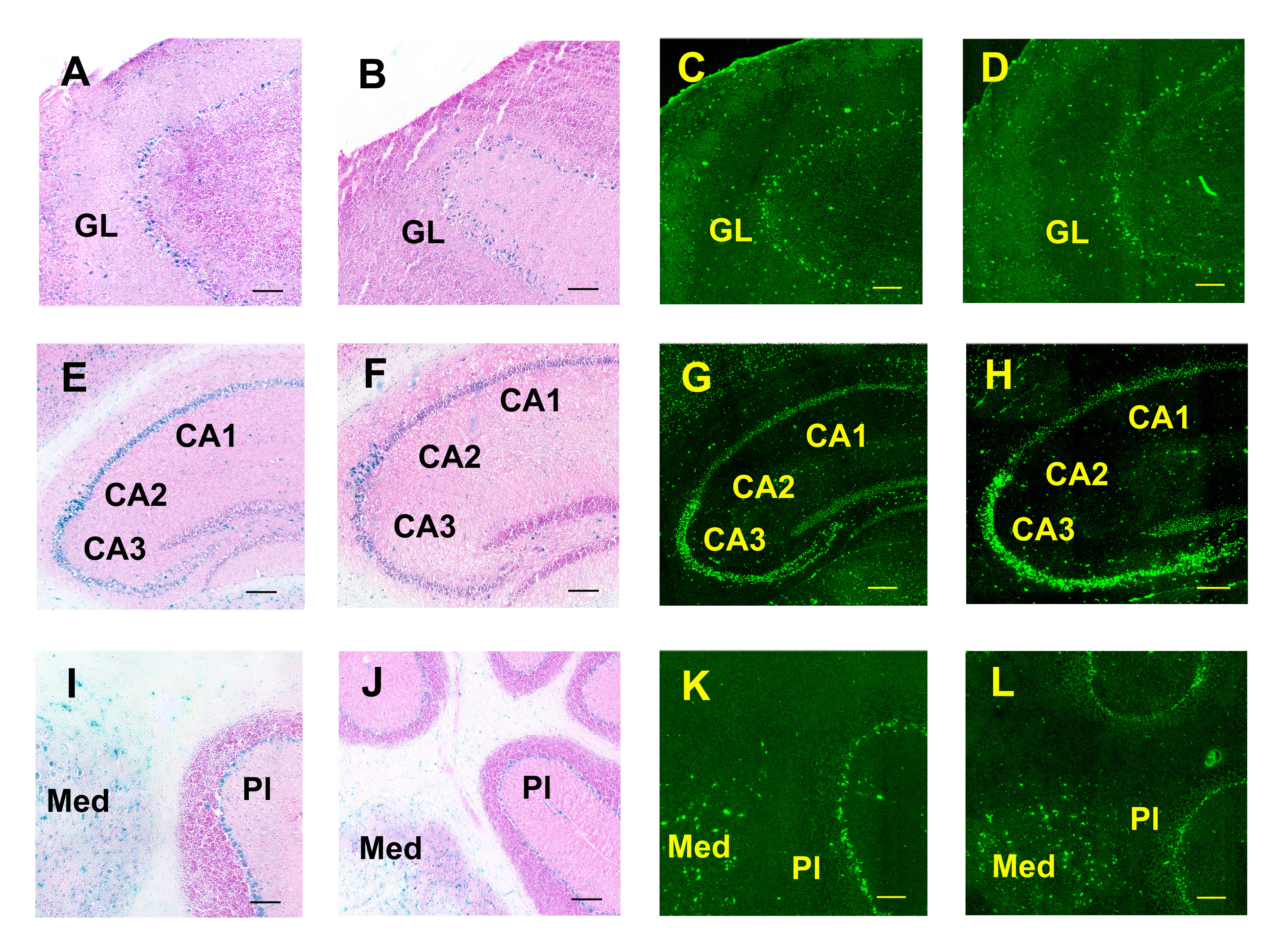

Supplement: Additional file 1 — Figure 1 – Comparison of tau/LacZ and tau/GFP reporter expression between two transgenic lines. The left two columns shows X-gal staining and the right two columns, GFP imaging. A, C, E, G, I and K are obtained from D13 line and are the same images shown in Fig. 5. A, C, M, O, Q and S, respectively. B, D, F, H, J and L are derived from D10 line. GL, glomerular layer of the olfactory bulb; CA1, field CA1 of hippocampus; CA2, field CA2 of hippocampus; CA3, field CA3 of hippocampus; Med, medial cerebellar nucleus; Pl, purkinje cell layer. Scale bar = 250 μm (A, C, E, G, I and K) or 50 μm (B, D, F, H, J and L). [file 1471-2199-7-41-S1.jpeg]
